# Input-dependent subcellular localization of spike initiation between soma and axon at cortical pyramidal neurons

**DOI:** 10.1186/1756-6606-7-26

**Published:** 2014-04-04

**Authors:** Rongjing Ge, Hao Qian, Na Chen, Jin-Hui Wang

**Affiliations:** 1 State Key Lab for Brain and Cognitive Sciences, Institute of Biophysics, Chinese Academy of Sciences, 15 Datun Road, Chaoyang District, 100101 Beijing, China; 2 University of Chinese Academy of Sciences, 100049 Beijing, China; 3 Qingdao University, Medical College, 38 Dengzhou, Shandong 266021, China

**Keywords:** Action potential, Soma, Axon, Neuron and sodium channel

## Abstract

**Background:**

Action potentials can be initiated at various subcellular compartments, such as axonal hillock, soma and dendrite. Mechanisms and physiological impacts for this relocation remain elusive, which may rely on input signal patterns and intrinsic properties in these subcellular compartments. We examined this hypothesis at the soma and axon of cortical pyramidal neurons by analyzing their spike capability and voltage-gated sodium channel dynamics in response to different input signals.

**Results:**

Electrophysiological recordings were simultaneously conducted at the somata and axons of identical pyramidal neurons in the cortical slices. The somata dominantly produced sequential spikes in response to long-time steady depolarization pulse, and the axons produced more spikes in response to fluctuated pulse. Compared with the axons, the somata possessed lower spike threshold and shorter refractory periods in response to long-time steady depolarization, and somatic voltage-gated sodium channels demonstrated less inactivation and easier reactivation in response to steady depolarization. Based on local VGSC dynamics, computational simulated spike initiation locations were consistent with those from the experiments. In terms of physiological impact, this input-dependent plasticity of spike initiation location made neuronal encoding to be efficient.

**Conclusions:**

Long-time steady depolarization primarily induces somatic spikes and short-time pulses induce axonal spikes. The input signal patterns influence spike initiations at the axon or soma of cortical pyramidal neurons through modulating local voltage-gated sodium channel dynamics.

## Introduction

Neurons integrate synaptic inputs and produce sequential spikes as digital codes in the brain. In terms of the sources of producing spikes, previous studies indicated that a single spike was initiated at axonal hillock [1-10], as well as somata and dendrites [11-21]. These data raise the question whether the location of spike initiation undergoes plasticity, in which input signals and local intrinsic properties may be involved. The elucidation to the dynamical alternation of spike initiation locations is critically important to understand how the neurons integrate synaptic signals and produce their digital codes in the brain efficiently.

Synaptic input signals that evoked neuronal spikes *in vivo* appeared long duration [15,22-25]. This long-time physiological depolarization induced sequential spikes dominantly at the somata [15], whereas short pulses initiated individual spikes at the axonal hillock [2,3,7,9]. Why the long-time versus short-time input signals influence the locations of spike initiation remains elusive. In addition, long-time signals integrated from synaptic inputs *in vivo* are classified into steady depolarization and fluctuated pulses [15]. It needs to be addressed whether these two patterns of input signals evoke sequential spikes at different subcellular compartments, an input-dependent plasticity of spike initiation location.

In terms of mechanisms underlying the subcellular localization of spike initiation, the dynamics and density of local voltage-gated sodium channel (VGSC) are presumably involved. It was suggested that a high density of VGSCs is critical for spike initiation at the axonal hillock [2,7,9]. However, the depolarization did not increase the number of functional VGSCs at axonal initial segment [26]. The intact initial segment with dense VGSC clusters was unnecessary for inducing spikes in the neurons [27]. VGSC’s dynamics also played an important role in spike initiation location [3,28]. Therefore, the changes in local VGSC dynamics and/or density are likely involved in the plasticity of spike initiation location. As the activity-dependent redistribution of high dense VGSCs occurred within axonal hillock and took a long time [29,30], the plasticity of spike initiation location may be based on local VGSC dynamics. In order to address the roles of input signal patterns and local VGSC dynamics in spike initiation relocation, we analyzed the sequential spikes and VGSC’s dynamics at the axonal bleb and soma of identical pyramidal neurons simultaneously in sensory cortical slices.

## Results

Membrane depolarization signals *in vivo* are long time, whose patterns are generally classified into steady and fluctuated pulses [15]. The action potentials can be evoked at various subcellular compartments, such as axonal hillock, soma and/or dendrite [2,3,11-13,15]. We have proposed to examine whether input signal patterns influenced spike-initiation location as well as how local VGSC dynamics regulated this input-dependent relocation of spike-initiation sites. In terms of the strategies to address these issues, we mainly analyzed correlations between input-signal patterns and spike-initiation locations by changing input signal patterns and subcellular compartment functions. This analysis would indicate whether the dynamic relocation of the spike-initiation sites was naturally present. To its underlying mechanisms, we focused on analyzing VGSC dynamics at these subcellular compartments in response to different input signals. Based on the dynamics of local VGSCs, we conducted computation simulation to test whether their dynamics characteristics were responsible for spike-initiation relocation. Furthermore, we examined whether the changes of local VGSC’s dynamics would shift spike-initiation location. Finally, we studied physiological impacts for the spike-initiation relocation, such as the efficacy of neuronal encoding.

### Input signal patterns influence spike-initiation at the somata and axons

If steady depolarization versus fluctuated one initiate sequential spikes at the different locations of given neurons, altering input signal patterns should drive spike-initiation relocation. In other words, if one of the subcellular compartments is preferentially sensitive to an input pattern for firing spikes, the spike capability in response to this input signal should be higher in this compartment than others. While changing input signal patterns, we analyzed the input–output curves of these compartments to assess their sensitivity to input signals and their spike capability.

**Figure 1:**
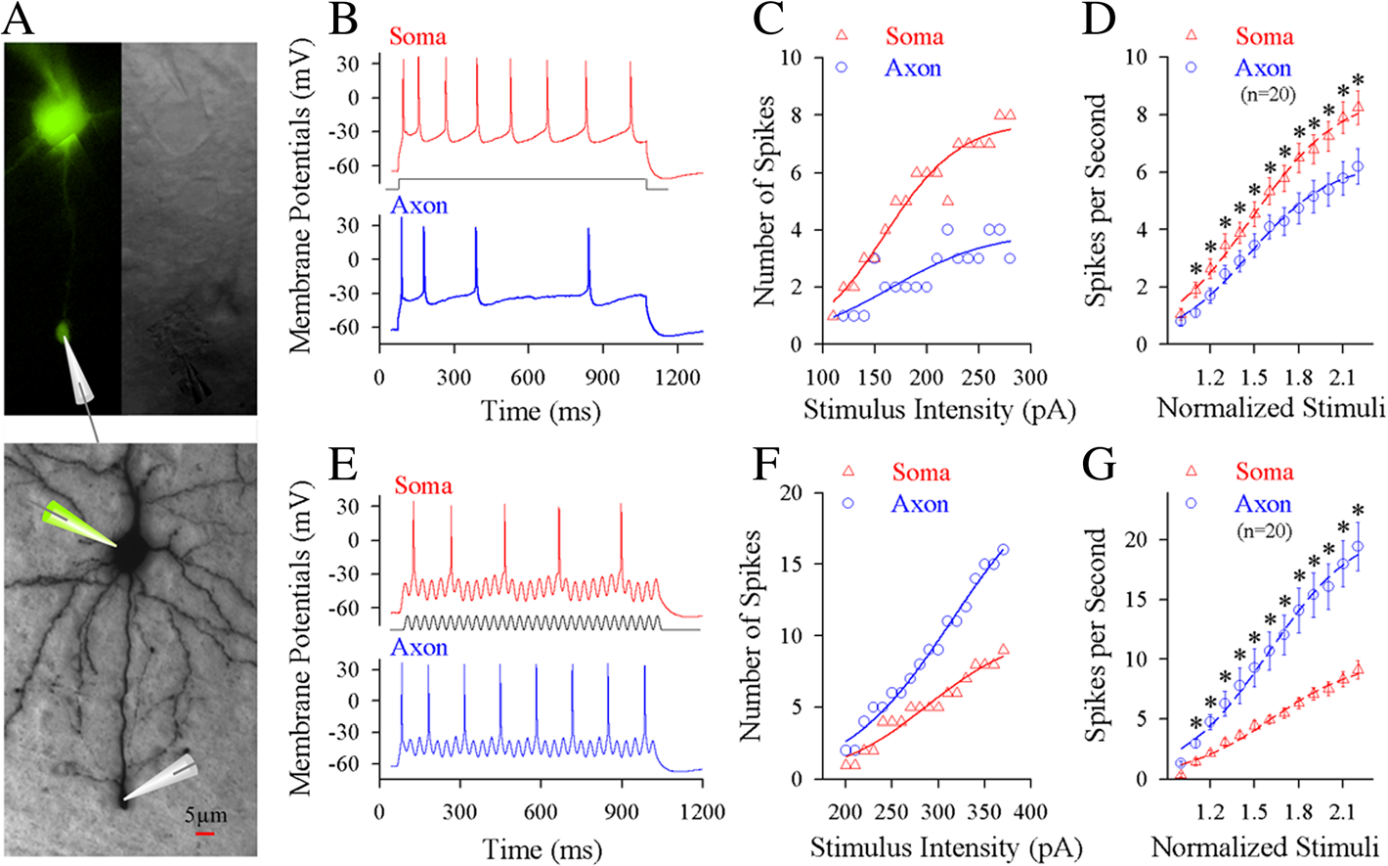
**Long-time step pulses initiate sequential spikes at the soma of cortical pyramidal neurons, but the fluctuated signals induce spikes at the axon. A)** Top panel shows the images of dual recording on the soma and axonal bleb under fluorescent (left)/DIC (right) microscope. Bottom panel shows an image of neurobiotin-labeled pyramidal neuron. **B)** Long-time step depolarization (middle black trace) induces more spikes at the soma (red trace) than at the axon (blue). **C)** shows the number of spikes vs. stimulus intensities at the soma (red symbols) and axon (blues) of this pyramidal neuron. **D)** illustrates input–output curves at the soma (red symbols) and axon (blues; n = 20, p < 0.05). **E)** The fluctuated signal (a cosine wave, middle black trace) induces more spikes at the axon (blue trace) than at soma (red). **F)** shows the ratio of spikes to cosine-waves vs. stimulus intensities at the axon (blue symbols) and the soma (reds) of this pyramidal cell. **G)** shows input–output curves for the axon (blue symbols) and the soma (reds; n = 20, p < 0.05).

Whole-cell recording was simultaneously conducted on the soma and axonal bleb of the same cortical pyramidal neurons to apply different input signals locally and to acquire sequential spikes (Figure 1A). Long-time (1 second) steady depolarization pulses or fluctuated pulses (cosine-wave, [31]) were injected into these two compartments to induce spikes, respectively (Additional file 1: Figure S1). While these depolarization pulses were injected into either of two compartments, the same amount of spikes was recorded at these two compartments. This spiking fidelity was due to the secure spike propagation on cell membrane between the axonal blebs and somata [13]. On the other hand, the subthreshold potential was electrotonically propagated and shunted by potassium channels, such that they would be decayed. We evaluated the capability of firing spikes (input–output and spike threshold) in these two compartments by injecting pulses and evoking spikes locally.

No matter what the axon or soma is a location to initiate spikes, this compartment should have higher capability to convert input signals into spikes, i.e., more efficient input-outputs transformation. Steady pulses in various intensities were injected into the soma and axonal bleb (20 ~ 50 μm away from the soma), respectively, to induce spikes (red trace in Figure 1B for the soma and blue for the axon). Figure 1C shows spikes per second versus stimulus intensities at the soma (red symbols) and axon (blue) in this example. Somatic input–output curve (red triangles in Figure 1D, n = 20) appears on the left-top side of axonal one (blue circles, n = 20; asterisks, p < 0.05, paired-t test), indicating that somatic spike threshold is lower and identical stimuli induce more somatic spikes. The somata are sensitive to long-time steady input signals.

On the other hand, the fluctuated pulses (black trace in Figure 1E) in various intensities were injected into the soma and axon of these pyramidal neurons, respectively, to induce spikes (red trace for the soma and blue for the axon). Axonal input–output curve (blue symbols in Figure 1F ~ G) is on left-top side of somatic one (red, n = 20; asterisks, p < 0.05, paired-t test), indicating that the axons are more sensitive to the fluctuated input signals than the somata.

**Figure 2:**
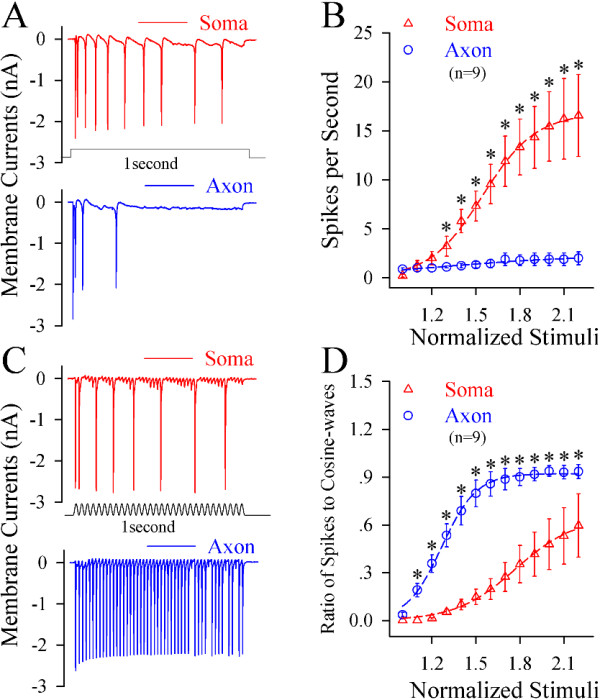
**Long-time step pulses preferentially initiate spikes at the soma of cortical pyramidal neurons under the condition of voltage-clamp recording, but the fluctuated signals induce spikes at the axon. A)** A long-time depolarization voltage (50 mV, middle black trace) induces more spikes at the soma (red trace) than at the axon (blue). **B)** illustrates input–output curves at the soma (red symbols) and the axon (blues; n = 9, p < 0.01). **C)** A fluctuated voltage signal (cosine-wave, 50 mV; middle black trace) induces more spikes at the axon (blue trace) than at the soma (red). **D)** shows input–output curves for the axon (blue symbols) and the soma (reds; n = 9, p < 0.01).

We examined these indications under a voltage-clamp to reduce the effect of passive membrane property on the recording. Steady depolarization induced more spikes at the soma (red trace in Figure 2A) than at the axon (blue). Somatic input–output curve (red triangles in Figure 2B) is on the left-top side of axonal one (blue circles, n = 9; asterisk, p < 0.05, paired-t test). On the other hand, the fluctuated signals induced more axonal spikes (blue trace in Figure 2C) than somatic spikes (red). Axonal input–output curve (blue circles in 2D) appears on left-top side of somatic one (red triangles, n = 9; asterisks, p < 0.05, paired-t test). The data are consistent with those under current-clamp (Figure 1).

**Figure 3:**
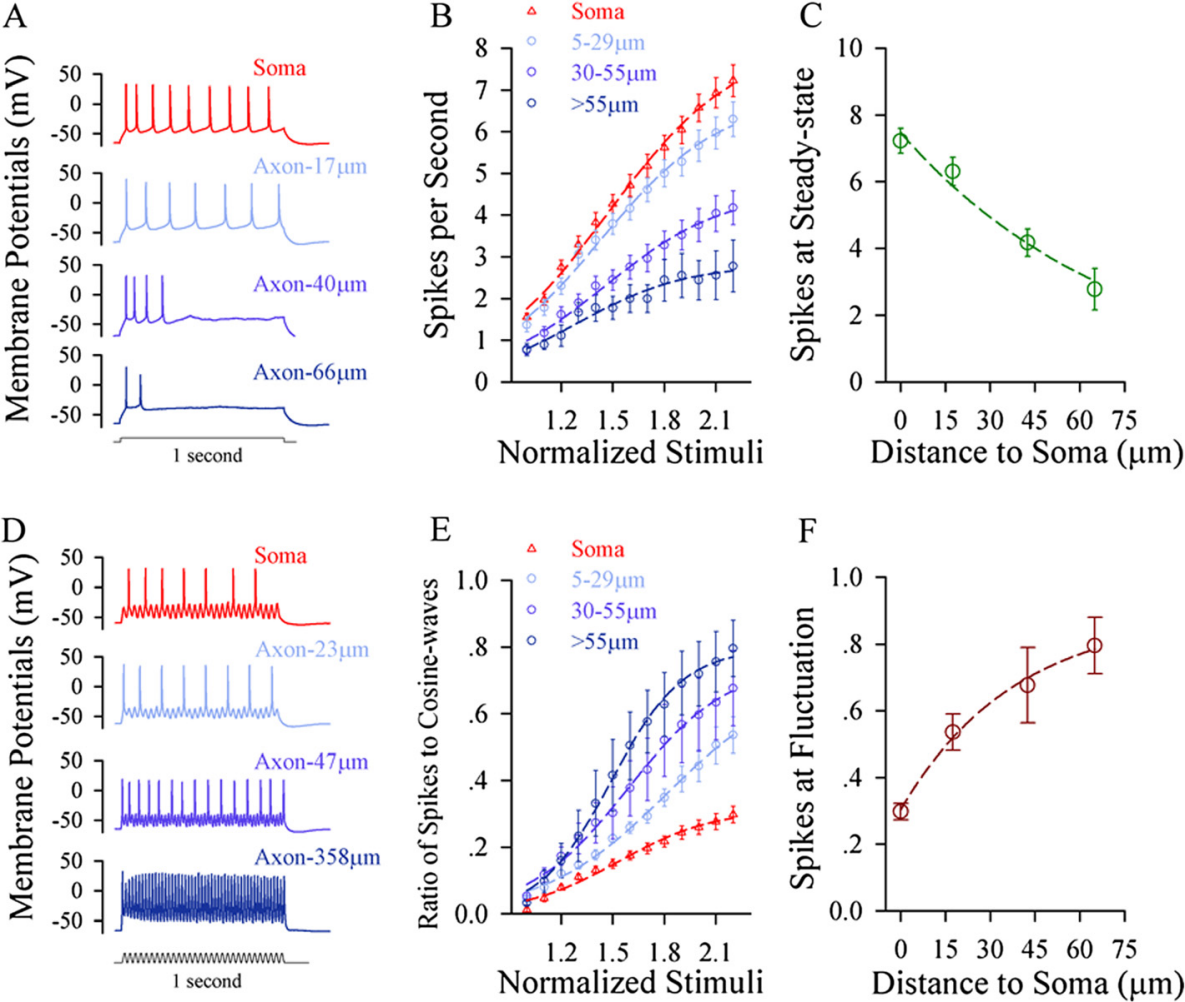
**The axon possesses the maximal ability of firing spikes in response to fluctuated signals, and the soma has the maximal ability of firing spikes in response to long-time steady depolarization. A)** An example shows spike waveforms from the soma to different axonal segments. **B)** shows the input–output curves for the soma (red symbols) and different axonal fragments in 5 ~ 29 μm (light-reds), 30 ~ 55 μm (pinks) and >55 μm (blues) away from the soma, in which sequential spikes are induced by long-time steady depolarization. **C)** illustrates the maximal values of input–output curves (normalized stimuli at 10) for steady-state pulses versus distances of axonal bleb to the soma. **D)** A sample shows spike waveforms from the soma to different axonal segments. **E)** shows input–output curves for the soma (red symbols) and distinct axonal fragments in 5 ~ 29 μm (light-reds), 30 ~ 55 μm (pinks) and >55 μm (blues) away from the soma, in which the spikes are induced by the fluctuated depolarization (cosine wave at 50 Hz). **F)** shows the maximal values of input–output curves for the fluctuated pulses vs. distances of axonal bleb to the soma.

If the soma is sensitive to steady pulse and the axonal segments are sensitive to fluctuated pulse, each of these compartments would fire the spikes in the highest frequency when receiving its sensitive signal. This assumption is based on a principle that sinoatrial node with the highest pacemaker spikes controls the heart rate. Steady depolarization pulses in various intensities were injected into the soma and different axonal segments, respectively, to induce spikes (Figure 3A). Figure 3B illustrates input–output curves for the somata (red symbols, n = 49) and axonal segments away from the soma in 5 ~ 29 μm (light-blue, n = 36), 30 ~ 55 μm (purple, n = 26) and >55 μm (dark-blue, n = 7). The maximal number of spikes from 3B versus the distances between axonal segments to soma were plotted in Figure 3C. The sensitivity to steady depolarization and the capability to produce its induced spikes are decreasing from the soma toward distal axons.

Moreover, the fluctuated pulses at various intensities were injected into the soma and different axonal segments, respectively, to induce spikes (Figure 3D). Figure 3E illustrates input–output curves at the soma (red symbols, n = 19) and the axonal segments away from the soma in 5 ~ 29 μm (light-blue, n = 13), 30 ~ 55 μm (purple, n = 10) and >55 μm (dark-blue, n = 10). The efficiency of converting the fluctuated signals into the spikes (the ratio of spikes to cosine-waves; dark-red) versus the distances of axonal segments to the soma were plotted in Figure 3F. The sensitivity to fluctuated signals and the capability to fire its induced spikes are increasing from the soma toward distal axons.

We analyzed input–output curve between the soma and axon segments of identical pyramidal neurons by altering input signal patterns. Long-time steady depolarization preferentially induces sequential spikes at the soma, but fluctuated one (sequential short-time pulses) initiates spikes at the axons. Moreover, we confirm this input-dependent plasticity of spike initiation location by changing compartment functions to shift spike initiation locations. It is noteworthy that spike initiation relocation is not caused by passive membrane property since there is no difference in the input resistance between the soma and axon (Additional file 2: Figure S2). As spike initiation is presumably controlled by VGSCs, we propose that the sensitivities of the somata and axons to the different input signals are due to the difference of their local VGSC’s dynamics, which we examined below.

### Somatic and axonal VGSCs are different in response to long-time and short-time signals

The VGSC dynamics sets spike capability as well as threshold potentials and refractory periods [32,33]. In examining the role of somatic and axonal VGSCs in this input-dependent plasticity of spike initiation location, we analyzed the influences of input signal patterns on spike thresholds and refractory periods at the soma versus the axon of pyramidal neurons. We also examined the influences of changing VGSC’s dynamics on spike thresholds and refractory periods at the soma and axon.

#### Somatic and axonal spike thresholds and refractory periods change in response to different signals

**Figure 4:**
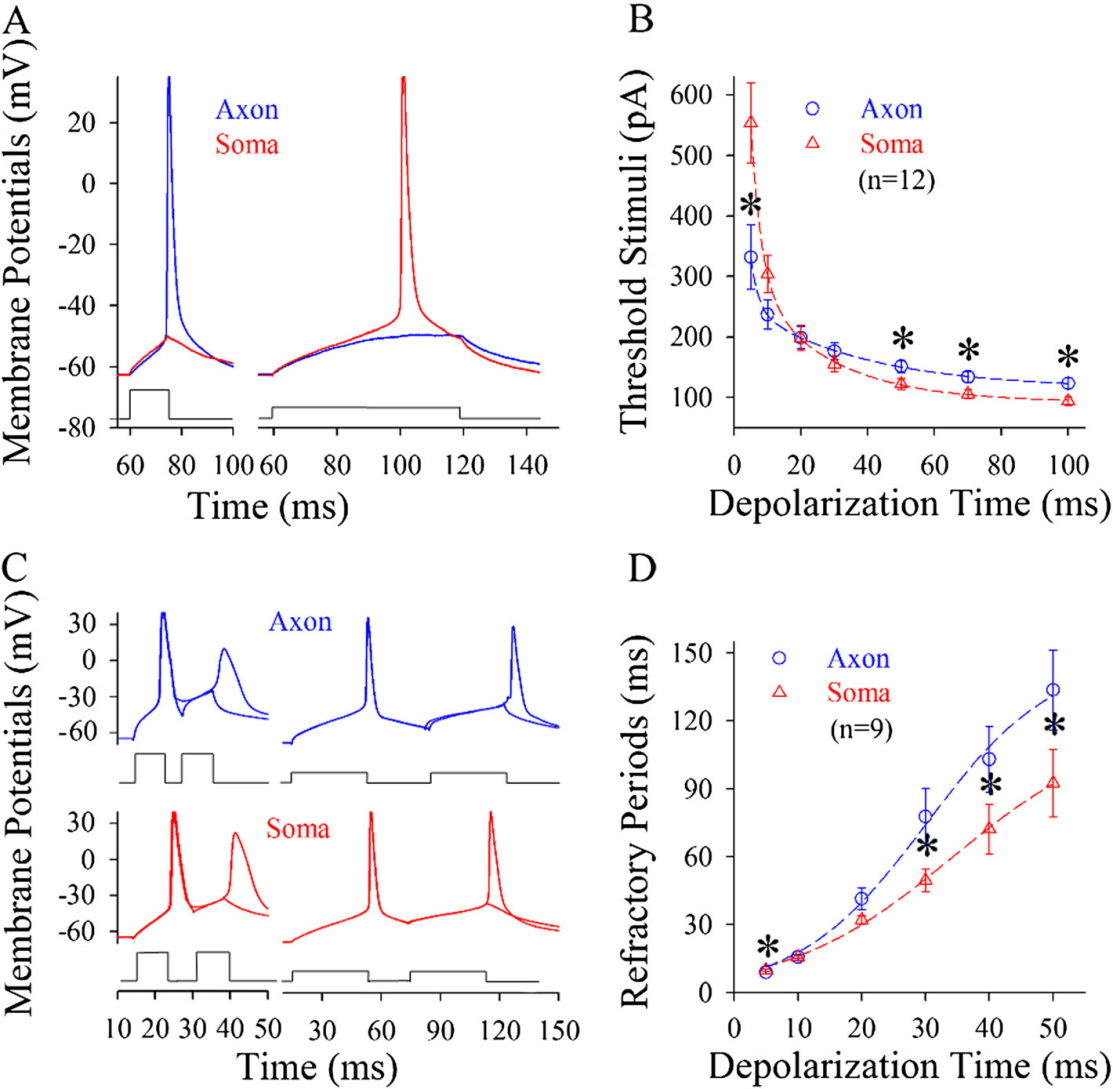
**Spike thresholds and refractory periods are changed dynamically by the patterns of input signals at the soma vs. axon. A)** Axonal spike thresholds appear low by short-time pulses (blue trace) and somatic ones are lower by long-time pulses (red). **B)** shows threshold stimuli vs. depolarization time at the soma (triangle symbols) and the axon (circles, n = 12). **C)** Left panels show that spike refractory periods appear short at the axon (blue traces at top panels) by short-time pulses. Right panels show that refractory periods are short at the soma (red lines at bottom panels) by long pulses. **D)** shows refractory periods vs. depolarization time at the soma (triangle symbols) and axon (circles, n = 9). Asterisks present p < 0.05.

Spike thresholds and refractory periods were measured at the soma and axonal bleb of identical cells [13]. In the measurements of somatic and axonal thresholds, pulse durations and intensities were inversely adjusted to induce the spikes at threshold level. Axonal spike thresholds appear low in response to short-time pulses (blue trace in Figure 4A), but somatic thresholds are low to long-time pulses (red). Threshold stimuli versus depolarization time at the soma (triangles) and the axon (circles) are plotted in Figure 4B, in which their fitting curves cross at 20 ms (asterisks, p < 0.05, n = 12). In addition, the refractory periods of axonal spikes induced by short-time pulses appear short (blue traces in Figure 4C), while those of somatic spikes by long-time pulses are short (reds). Figure 4D shows spike refractory periods versus depolarization time at the axon (circles) and soma (triangles, n = 9; p < 0.05). When long-time depolarization signals are inputted to pyramidal neurons, somatic spike thresholds and refractory periods are converted to be lower than axonal ones, so that sequential spikes are initiated primarily at cell body, or vice versa.

**Figure 5:**
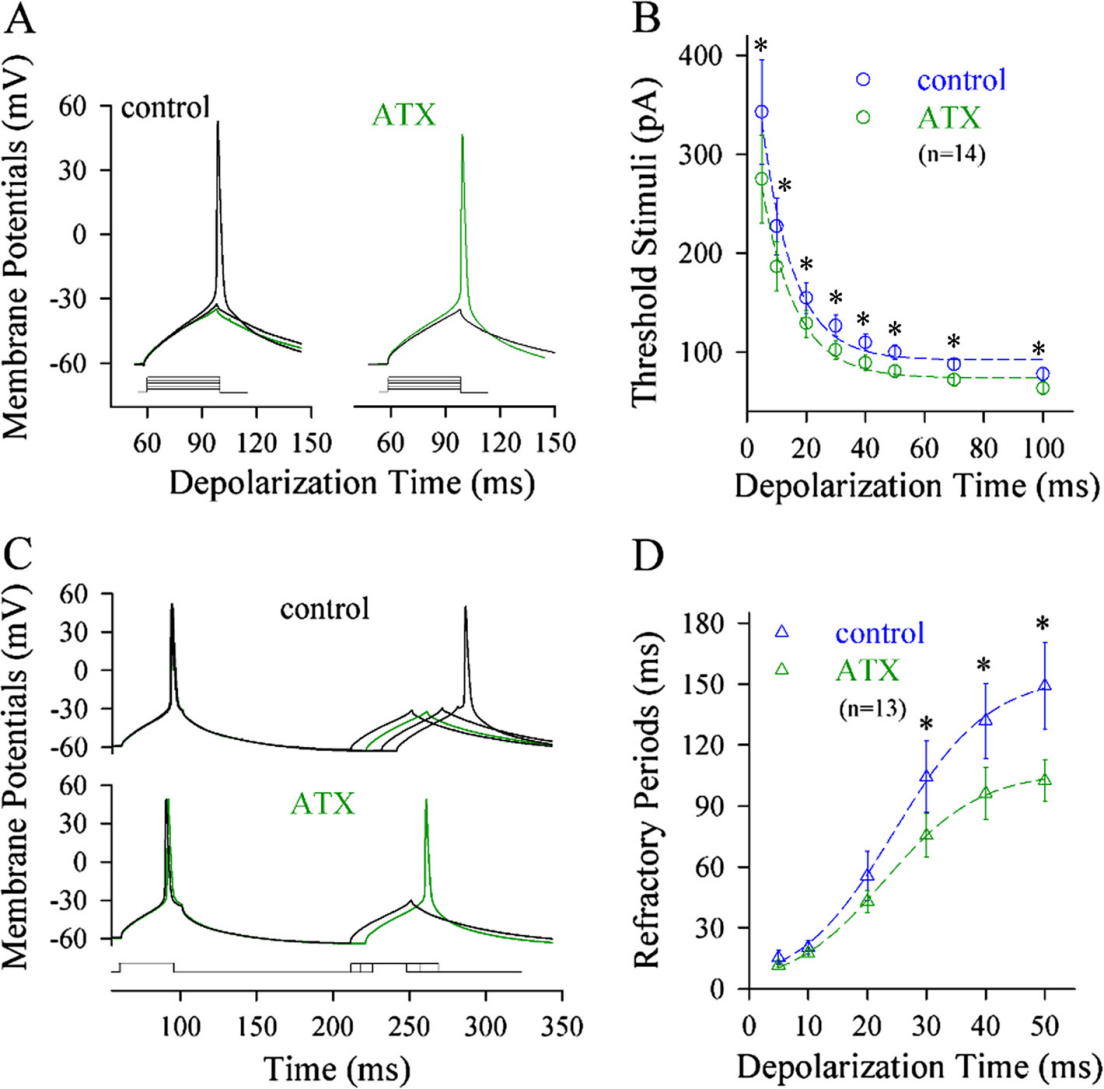
**ATX, a reagent of preventing VGSC inactivation, reduces spike thresholds and shortens refractory periods at the axon.** Spike thresholds and refractory periods are measured dynamically by changing the patterns of input signals at the axon. **A)** shows the effect of ATX on axonal spike thresholds (green traces). **B)** shows threshold stimuli vs. depolarization time at the axon. ATX reduces axonal spike thresholds significantly (green symbols; asterisks, p < 0.05, n = 14; paired t-test). **C)** shows the effects of ATX on the refractory periods of axonal spikes (green trace). **D)** illustrates spike refractory periods vs. depolarization time at the axon. ATX shortens the refractory periods of axonal spikes induced mainly by long-time pulses (green symbols, n = 13; asterisks, p < 0.05).

We also examined the effects of reducing VGSC inactivation on spike threshold and refractory period. The reduction of VGSC inactivation was fulfilled by using anemone toxin (ATX), a blocker of VGSC inactivation [34,35]. ATX appears to reduce axonal spike threshold (green traces in Figure 5A). Figure 5B illustrates threshold stimulations versus depolarization time at the axon, in which the spike thresholds are attenuated by ATX (green symbols; p < 0.05, n = 14). Furthermore, ATX appears to shorten axonal refractory periods (green trace in Figure 5C). Figure 5D shows spike refractory periods versus depolarization time at the axon, in which ATX shortens spike refractory periods mainly induced by long-time pulses (green symbols; p < 0.05; n = 13). It is noteworthy that ATX does not influence somatic spike threshold and refractory period (Additional file 3: Figure S3). These data indicate that long-time depolarization signals mainly inactivate axonal VGSCs and lower its spike capability.

In terms of the influence of input signals on VGSC’s dynamics, long-time steady depolarization mainly inactivates axonal VGSCs, such that the steady signals initiate sequential spikes at the somata and the fluctuated signals to initiate spikes at the axons. We further investigated how long-time steady depolarization pulses influence the inactivation and reactivation of axonal VGSCs vs. somatic ones.

#### Axonal VGSCs are easily inactivated and less reactivated by long-time steady depolarization

**Figure 6:**
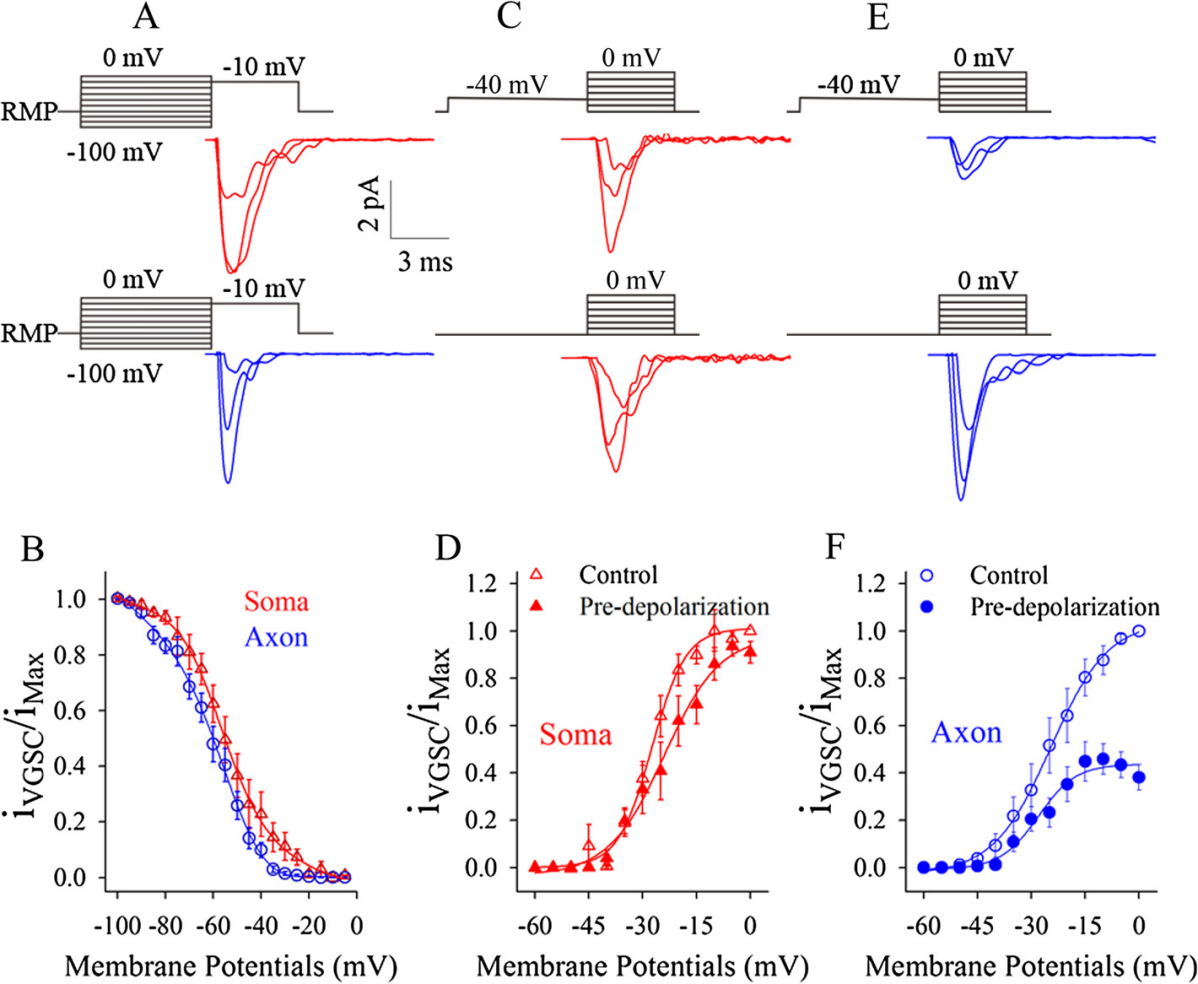
**Pre-depolarization inactivates voltage-gated sodium channels (VGSC) partially and weakens their subsequent activation at the axon. A-B)** Pre-depolarization pulses inactivate VGSCs mainly at the axons (A). The inactivation curve of axonal VGSCs (blue symbols in B) is on the left side of that of somatic ones (red triangles). Axonal VGSCs are more easily inactivated. **C-D)** A pre-depolarization pulse weakens somatic VGSCs activation slightly (top panel in C for waveform and red-filled symbols in D) compared with control (bottom panel in C and opens in D). **E-F)** A pre-depolarization pulse significantly weakens axonal VGSCs activation (top panel in E for waveform and blue-filled symbols in F) compared with control (bottom in E and opens in F).

VGSCs were inactivated in voltage-/state-dependent manners [36,37]. *In vivo* signals induced sequential spikes on the long-time depolarization [13,22-25]. This pre-depolarization may inactivate axonal VGSCs [38] to elevate spike threshold and refractory period (Figure 4), such that spike initiation shifts to the soma [15,39]. To examine this implication, we compared VGSC inactivation and reactivation at the soma versus axon by cell-attached recording simultaneously at the two subcellular compartments of identical pyramidal neurons (Figure 6).

VGSCs’ inactivation curves were measured by giving pre-depolarization pulses ahead of a fixed depolarization pulse. Compared to somatic VGSCs (red traces in Figure 6A), axonal ones (blue trace) appear obvious inactivation in response to these pre-depolarization pulses. Axonal VGSC inactivation curve (blue line/circles in Figure 6B) is on the left side of somatic one (red triangles), that is, the pre-depolarization pulses make axonal VGSCs to be easily inactivated. This result is consistent to a report that axonal Nav1.6 inactivation is more than somatic Nav1.2 inactivation during pre-depolarization [40].

To test whether the inactivation of axonal or somatic VGSCs affects their subsequent activation, we measured their activation curves under a pre-depolarization. Figure 6C ~ D shows somatic VGSC’s activation with (top panel in 6C and filled triangles in 6D) and without a pre-depolarization (control; bottom in 6C and open triangles in 6D). Figure 6E ~ F shows axonal VGSC’s activation under partial inactivation (top panel in 6E and filled circles in 6F) and control (bottom in 6E and open circles in 6F). Axonal VGSCs become difficultly reactivated if a pre-depolarization induces their partial inactivation.

In summary, long-time signals dominantly suppress the reactivation of axonal VGSCs and lead to the high values of axonal spike thresholds and refractory periods (Figures 4, 5 and 6). So, long-time steady signals mainly initiate sequential spikes at the soma, and the fluctuated ones induce spikes at the axon (Figures 1, 2 and 3). We examined this indication by changing axonal VGSC dynamics and computational simulation.

### The role of local VGSCs in input-dependent relocation of spike initiation

The data above indicate that spike onset at the axon or the soma is related to their local VGSC dynamics. To examine the role of local VGSCs in the input-dependent plasticity of spike initiation location, we conducted computational simulation as well as changed local VGSC dynamics to see spike initiation relocation. Introducing VGSCs, which possess somatic featured VGSC dynamics, into computational modeling is expected to simulate sequential spikes similar to somatic spikes, or vice versa. The experimental upregulation and downregulation of local VGSC dynamics are expected to cause the relocation of spike initiation.

#### Easier inactivation and less reactivation of axonal VGSCs simulate spikes being somatic origin

**Figure 7:**
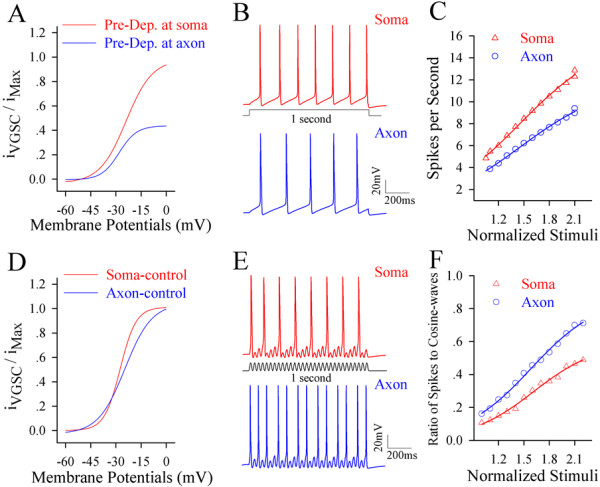
**Computational simulation favors a somatic origin of sequential spikes under the condition of pre-depolarization, and axonal origin for spikes without pre-depolarization. A)** A pre-depolarization pulse significantly inactivates axonal VGSCs, including an increase of their threshold by ΔV and a decrease of activation by Δi. **B)** The number of simulated spikes appears higher at the soma (red trace) than the axon (blue) induced by a long-time step depolarization (black). **C)** illustrates spike number vs. normalized stimuli at the soma (red triangles) and the axon (blue circles; n = 20, p < 0.01). **D)** shows the activation curves of somatic VGSCs (red trace) and of axonal ones (blue) by the pulses without pre- depolarization and hyperpolarization. **E)** The number of simulated spikes appears higher at the axon (blue trace) than the soma (blue) induced by a cosine-wave depolarization pulse (black). **F)** illustrates input–output curves at axonal spikes (blue circles) and somatic ones (red triangles; n = 20, p < 0.01).

In computational simulation, we introduced the curves of axonal VGSC reactivation (blue trace in Figure 7A) and somatic one (red) under pre-depolarization (from Figure 6D and 6F) into *NEURON* model [41,42]. In the modeling, threshold potentials (ΔV) for axonal VGSCs were more positive than somatic VGSCs, and axonal VGSC reactivation (Δi) was 50% lower than somatic one. By introducing these factors, long-time steady signal induced more simulated spikes at the soma (red trace in Figure 7B) than the axon (blue). The threshold of sequential spikes is lower, and the number of spikes by identical stimuli is higher at the soma (red symbols in Figure 7C) than the axon (blue).

On the other hand, we inputted the curves of axonal VGSC activation (blue curve in Figure 7D) and somatic one (red) under no pre-depolarization (control in Figure 6D and F) into *NEURON* model. In the modeling, threshold potential (ΔV) and VGSC activation (Δi) for the axon were similar to those for the soma. The fluctuated signals under this condition induced more simulated spikes at the axon (blue trace in Figure 7E) than the soma (red trace). The thresholds of sequential spikes are lower, and the number of spikes by identical stimulus is higher at the axon (blue symbols in Figure 7F) than the soma (red).

The data from computational simulation supports the experimental data that long-time steady depolarization mainly inactivates axonal VGSCs and initiates sequential spikes at the soma, whereas fluctuated signals initiate the spikes at the axon, i.e., input-dependent plasticity of spike initiation location.

#### The manipulation of axonal VGSC’s function changes the location of spike initiation

**Figure 8:**
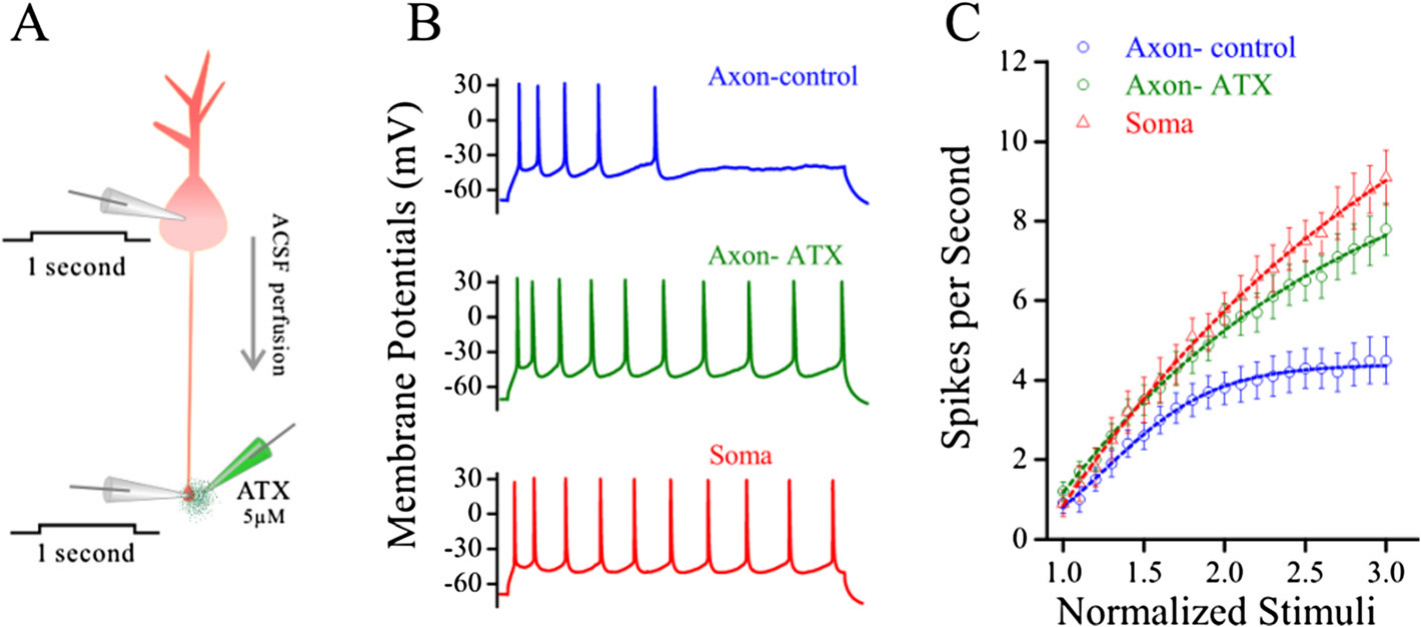
**ATX dominantly secures the axonal capability of firing sequential spikes induced by long-time steady depolarization pulses. A)** shows a diagram for whole-cell recording at the soma and axon, and puffing 5 μM ATX (green pipette) toward axonal bleb. **B)** Long-time steady pulse initiates sequential spikes at the axon of cortical pyramidal neurons before (blue trace) and after ATX application (green), as well as sequential spikes at the soma of pyramidal neurons (red trace). **C)** shows spikes per second versus stimulus intensities at the axons of pyramidal neuron before (blue circles) and after ATX applications (greens, n = 10; asterisks, p < 0.05), as well as input–output curves at the somatic recordings (red triangles; n = 10).

Long-time steady depolarization initiates more somatic spikes than axonal spikes (Figures 1, 2 and 3) by inactivating axonal VGSCs (Figures 4, 5, 6). If it is right, axonal spike capability in response to steady depolarization should be enhanced by upregulating axonal VGSC dynamics. While puffed ATX to the axonal blebs of pyramidal neurons (a green electrode in Figure 8A), we observed that ATX increased the number of spikes induced by steady pulses at the axon (Figure 8B). Figure 8C shows input–output curves for the soma (red symbols) as well as axon before (blues) and after using ATX (greens; p < 0.01, n = 10). The prevention of axonal VGSC inactivation strengthens axonal spike capability.

**Figure 9:**
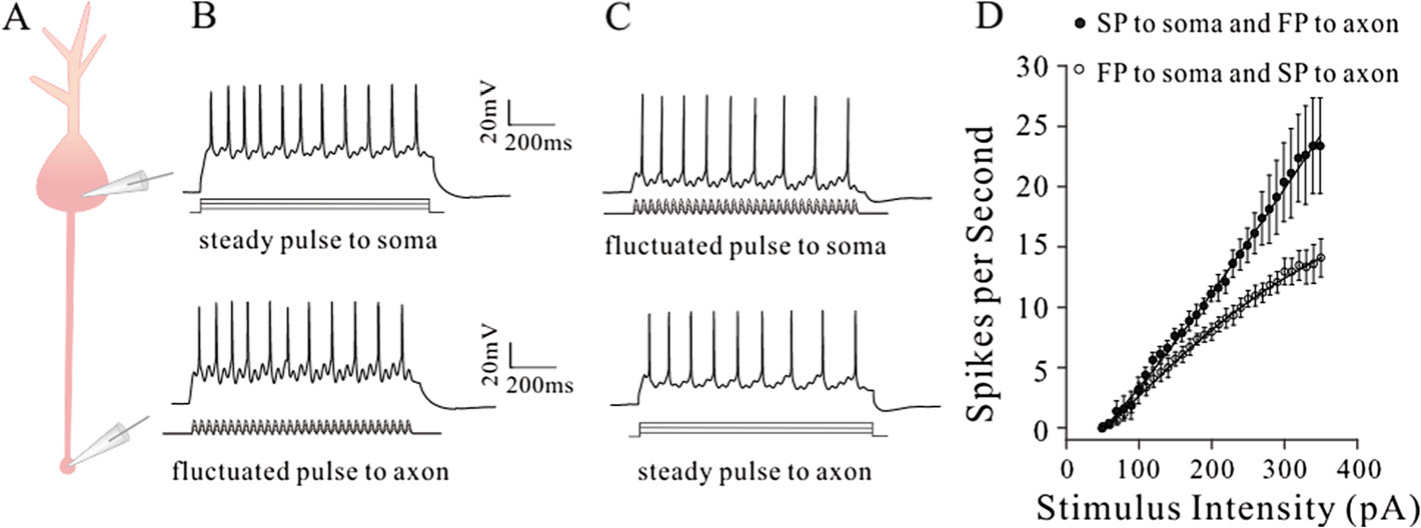
**The reduction of axonal VGSC function by steady depolarization signal may weaken axonal capability to produce spikes in response to fluctuated signals.** Axonal VGSCs are inactivated by long-time steady depolarization pulses in two ways. **A)** shows dual recording at the soma and axonal bleb. **B)** The fluctuated pulse (FP) is injected into the axon to induce spikes, while the steady pulse (SP) is injected into the soma to indirectly inactivate axon VGSCs. **C)** FP is injected into the soma to induce the spikes, while SP is injected into the axon to directly inactivate axonal VGSCs. **D)** shows input–output curves for directly (open symbols) and indirectly (filled symbols) inactivating axonal VGSCs. Spike capability induced by the fluctuated pulse is more dominantly reduced by the direct inactivation of axonal VGSCs than the indirect inactivation of axonal VGSCs does (n = 7).

On the other hand, we inactivated axonal VGSCs by long-time depolarization pulse to examine whether axonal spike capability in response to fluctuated signal would be weakened. In dual recording on the soma and axonal bleb of identical pyramidal cells (Figure 9A), we inactivated axonal VGSCs by using long-time steady depolarization pulses in two methods. The fluctuated signals were injected into the axons to evoke the spikes, while the steady signals were injected into the somata to indirectly inactivate axonal VGSCs (Figure 9B). The fluctuated signals were injected into the somata to evoke the spikes, while the steady signals were injected into the axons to directly inactivate axonal VGSCs (Figure 9C). Both approaches reduce spike capability induced by the fluctuated signal, but the direct inactivation of axonal VGSCs more dominantly reduces spike capability (gray symbols in Figure 9D) than the indirect inactivation of axonal VGSCs does (orange symbols, n = 7). Neuronal spike capability and patterns by inactivating axonal VGSCs are similar to somatic spikes (please compare to Figure 3). Thus, an inactivation of axonal VGSCs weakens axonal spike capability in response to the fluctuated signals, or shifts spike initiation location toward the soma.

### The input-dependent plasticity of spike initiation location increases neuronal encoding efficiency

**Figure 10:**
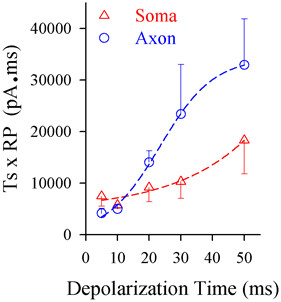
**The cortical pyramidal neurons encode digital spikes in an economical way.** The plot shows the comparison of the energetic barriers for the soma and axon to fire spikes induced by the different durations of synaptic inputs. The values of energetic barriers are calculated by multiplication of threshold and refractory period. These values at the soma are low by giving long-time pulses (red symbols) and the values at the axon are low by short ones (blues).

In terms of physiological significances for the plasticity of spike initiation location, we assume that this relocation of spike initiation may be for the neurons to save the energy during neuronal encoding. The neurons have to step over two barriers [43], spike thresholds and refractory periods [32,44-46], to produce the spikes. If this assumption is right, we should see lower energetic barriers for somatic spikes by long-time steady signal or axonal spikes by fluctuated signal. By calculating the energetic barrier, the multiplication of spike threshold and refractory period [43], we found that this value at the soma was low by using long-time pulses (red symbols in Figure 10) and the value at the axon was lower by short-time ones (blues). Thus, spike initiations at the soma by long-time signals and at the axon by short ones make the neurons to utilize the lower levels of energy. In other words, the input-dependent plasticity of spike initiation location is beneficial for the neurons to save energy during encoding digital signals.

In addition, the portion of the fluctuated signal was about 40% in the integrated synaptic signals [15]. To convert synaptic inputs into spikes efficiently, the neurons should make each of fluctuated signals to induce a spike. Where is this optimal conversion fulfilled? As the axon is more sensitive to the fluctuated signal (Figures 1, 2 and 3), it is likely a subcellular compartment that converts the fluctuated signals into the spikes with an optimal match in their frequencies. We tested this possibility by measuring the optimal efficiency of converting cosine waves into spikes at the soma and the axonal segments.

**Figure 11:**
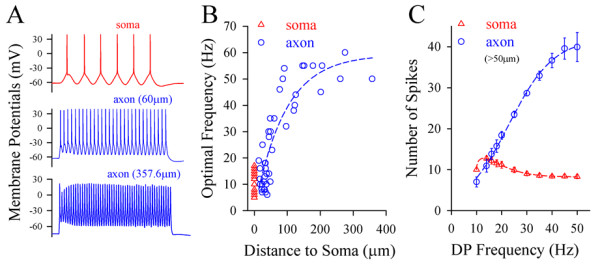
**The axons confer the pyramidal neurons to efficiently encode the digital spikes in response to the fluctuated input signals.** The fluctuated signals (cosine waves) at threshold stimuli in different frequencies were injected into the soma and different axonal segments, respectively, to induce spikes. **A)** shows the optimal frequency of converting the fluctuated signals into spikes at the soma and axons. **B)** illustrates optimal frequency versus distance of axonal segments to the soma (soma, n = 17; axons, n = 43). **C)** shows the frequency of depolarization (DP) vs. number of spikes (n = 11). Axonal segments away from the soma larger than 50 μm appear a subcellular compartment of converting the fluctuated signals into the spikes with optimal match in their frequencies.

The fluctuated signals (cosine wave) with frequency increments were injected into the soma and different axonal segments, respectively, to induce the spikes. If the soma or axon in response to these signals reached an optimal level (each of pulses triggered a spike), we named it as optimal response frequency. The optimal frequency of converting fluctuated signals into spikes appears the highest at the distal axon (Figure 11A). Figure 11B shows optimal frequency versus distance of axonal segments to the soma (n = 17 for soma; n = 43 for axon). Figure 11C shows number of spikes versus frequency of depolarization (DP) at the axon (blue symbols) and soma (reds, n = 11). Therefore, the axonal segments away from the soma above 50 μm are the subcellular compartment that converts the fluctuated signals into the spikes with optimal match in high frequencies, i.e., the axons confer the neurons to efficiently encode digital spikes in response to the fluctuated input signals.

## Discussion

Long-time steady depolarization initiates sequential spikes dominantly at the somata of cortical pyramidal neurons, whereas fluctuated signal instigates the spikes at their axons (Figures 1, 2 and 3), i.e., an input-dependent plasticity of spike initiation locations. In terms of its mechanism, long-time pulses make somatic spike thresholds and refractory periods to be lower and short-time ones make such parameters to be lower at the axon (Figures 4 and 5). Long-time depolarization mainly inactivates axonal VGSCs, or vice versa (Figure 6). Computational simulation based on the kinetics of axonal and somatic VGSCs supports that long-time steady depolarization induces somatic spikes and fluctuated signals do axonal ones (Figure 7). Our studies in the experiments and computational simulation reveal the spike initiation relocation between the somata and axons of cortical pyramidal neurons, which is controlled by local VGSC kinetics.

In order to make sure the input-dependent plasticity of spike initiation location, we applied multiple strategies. For instance, subcellular compartments in terms of their sensitivity to distinct input signals and their ability to produce spikes were analyzed under the conditions of current-clamp and voltage-clamp recordings. These experiments were conducted from the somata to different axonal segments in order to find a dominant location of spike initiation. Moreover, the methods in the functional upregulation and downregulation of subcellular compartments were used to analyze the relocation of spike initiation. The results proved by these strategies make the conclusion, the input-dependent plasticity of spike initiation location, to be convincing.

Current reports indicated that the neuronal activities induced the plasticity in terms of the length and distribution of high dense VGSCs at axonal hillock [29,30]. This plasticity of VGSC distribution spends long time for its onset and occurs within axonal hillock. Different from this slow onset plasticity, the input-dependent plasticity of spike initiation location in our study develops quickly, shifts between the soma and axon (Figures 1, 2 and 3) and depends on local VGSC kinetics (Figures 4, 5 and 6). This subcellular relocation of spike initiation is a novel type of neuronal plasticity, compared to long-term plasticity in neuronal excitability [47-54], neuronal homeostasis [55] and VGSC redistribution [29,30].

A major finding in our studies is that long-time steady depolarization signals initiate sequential spikes at the somata of cortical pyramidal neurons (Figures 1, 2 and 3) through inactivating axonal VGSCs and conferring somatic VGSCs to be less inactivation and easily reactivation (Figures 4, 5 and 6). As long-time steady pulses are a major portion of *in vivo* signals [15], a somatic origin to fire sequential spikes is physiologically important. One could argue that the smaller axonal volume than somatic one plus the higher density of axonal VGSCs may enable the depolarization to bring denser positive charges into the axon that facilitates the axons to reach a threshold and produce the first spike ahead of the soma. However, the voltage-/state-dependent VGSC inactivation (Figures 4, 5 and 6; [36,37]) may also make this depolarization inactivating axonal VGSCs, especially axonal Nav1.6 [40], which does not help to fire sequential spikes at the axon.

On the other hand, the fluctuated depolarization signals, a sequence of short-time pulses, initiate axonal spikes at cortical pyramidal neurons (Figures 1, 2 and 3), due to low threshold and refractory periods (Figure 4) mediated by VGSCs (Figures 5 and 6). Our data grant a dogma that short-time pulse initiates a single spike at axonal hillock [1,4,6], due to low thresholds and high density of VGSCs at this segment [2,3,7,9,10,56-67]. It is noteworthy that the durations of *in vivo* fluctuated signals are over 50 ms [15] and unitary synaptic events last for longer than 20 ms [13,68]. Despite a high density of VGSCs at axonal hillock, depolarization pulses above these durations make the number of functional VGSCs not being high at AIS [26] as well as convert somatic threshold and refractory periods to be lower (Figure 4), such that physiological signals induce sequential spikes being somatic in origin [15].

What are physiological impacts for the input-dependent plasticity of spike initiation location? This plasticity is related to saving the energy during neuronal encoding. The neurons step over two barriers [43], spike threshold and refractory period [32,44-46], to produce the spikes. The energetic barriers to fire the spikes at the soma are lower by giving long-time pulses, and these values at the axon are lower by short-time ones (Figure 10). Therefore, the relocation of spike initiation is used for the neurons to save the energy in spike encoding. Moreover, the propagation of soma-integrated input signals in long-time depolarization toward the axons may inactivate their VGSCs and be shunted by GABAergic receptor-channels at axonal hillock. To prevent the decay of these signals in their propagation, the integration of analog synaptic signals and the encoding of digital spikes are better fulfilled in a single subcellular compartment, i.e., neuronal soma. On the other hand, fluctuated pulses allow VGSCs to be recovery (Figure 4) and even facilitate Nav1.6 activation at the axons [69], such that the axons serve to an optimal match for the frequency between input signals and spikes as well as optimal response to high frequent inputs (Figures 1, 2, 3 and 11).

We have studied the regulation of spike initiation relocation between the soma and axon of cortical pyramidal neurons, a role of VGSC dynamics in this input-dependent plasticity and its physiological impact for the neurons to save energy. The studies in these three aspects reveals the natural features of spike initiation and strengthens the local presence in the input-dependent relocation of spike initiation.

## Methods and materials

### Brain slices

The study and all experiments were fully approved by the Institutional Committee of Animal Care Unit in Administration Office of Laboratory Animals Beijing China (ID# B10831). The slices from sensory cortices (300 μm) were prepared from FVB mice. Mice in postnatal day 15–20 were anesthetized by injecting chloral hydrate (300 mg/kg) and decapitated with a guillotine. The cortical slices were cut with a Vibratome in the modified and oxygenized (95% O_2_/5% CO_2_) artificial cerebrospinal fluid (mM: 124 NaCl, 3 KCl, 1.2 NaH_2_PO_4_, 26 NaHCO_3_, 0.5 CaCl_2_, 5 MgSO_4_, 10 dextrose and 5 HEPES; pH 7.35) at 4°C, and then were held in the normal oxygenated ACSF (mM: 126 NaCl, 2.5 KCl, 1.25 NaH_2_PO_4_, 26 NaHCO_3_, 2.0 CaCl_2_, 2.0 MgSO_4_ and 25 glucose; pH 7.35) 35°C for 1 hour before the experiments. A slice was transferred to a submersion chamber (Warner RC-26G) that was perfused with normal ACSF for electrophysiological experiments [70].

### Dual recording

The soma and axonal bleb [71] of identical pyramidal cells in layers IV-V of cerebral cortex were simultaneously recorded (MultiClapm-700B, Axon Instrument Inc. CA USA) under a fluorescent/DIC microscope (Nikon FN-E600). The identification of axonal blebs rather than dendritical bleb was based on the diameter and branches of processes as well as the polarity of neurons [13,15]. Neuronal processes with less branches and fine diameter are axon. The electrical signals were inputted into pClamp-10 (Axon Instrument Inc. USA) with 50 kHz sampling rate.

In whole-cell recording on the soma and axonal bleb, the action potentials were induced at these two compartments, respectively, by long-time steady depolarization pulses or cosine waves in various intensities, but the stimulus intensities to the soma and axonal bleb of each neuron were identical. As the excitability varied among the neurons [68], the stimulus intensities for different cells were normalized, in which we set the threshold current as 1, and then increased pulse intensities by 10% gradually, i.e., 1, 1.1, 1.2……2.2 times of threshold current. This strategy made the data from different neurons to be statistically comparable. A judgment for recording two sites from an identical neuron was based on the presence of direct and corresponding electrical signals. Transient capacitance was compensated and output bandwidth was 3 kHz. Pipette solution contained (mM) 150 K-gluconate, 5 NaCl, 0.4 EGTA, 4 Mg-ATP, 0.5 Tris- GTP, 4 Na-phosphocreatine and 5 HEPES (pH 7.4 adjusted by 2 M KOH). The osmolarity of pipette solution freshly made was 295–305 mOsmol, and the pipette resistance was 10–15 MΩ.

It is noteworthy that the axonal blebs of pyramidal neurons were formed from the resealing at the end of cut axons during slice preparation. Although this preparation could be argued as an injured axon, several lines of evidence indicated the functional intact of axonal blebs. The values of resting membrane potentials and action potentials are closely normal (Figures 1, 2, 3, 4 and 5). These axons underwent the functional plasticity, i.e., upregulation and downregulation in their abilities to fire spikes (Figures 8 and 9). Moreover, other studies in immunohistochemistry and electrophysiology suggested that the functions of axonal blebs were likely normal [7].

The intrinsic properties of the somata and axonal blebs in our studies included spike thresholds and refractory periods measured under a whole-cell current-clamp. Spike thresholds were measured by depolarization pulses with the inverse changes in their intensities and durations. Spike refractory periods were measured by injecting two pulses (5% above threshold) with various durations into the neurons after each spike, in which inter-pulse intervals were adjusted [32,33,55,68,72]. In the measurement of spike refractory periods versus pulse durations, the increment of depolarization duration was inversely associated with the reduction of its intensities, which were 5% above thresholds.

### Pharmacological studies

Anemone toxin (ATX; [34] was dissolved freshly in the standard ACSF at 5 μM for a final concentration, and was given to a specific subcellular compartment by a pipette perfusion via the pressure. In the application of ATX, fluorescent Alex-488 (10 μM) was also added into this solution in order to show the size of areas for perfusing ATX to be restricted to specific subcellular compartment. The pressure was added into the pipettes to have ATX/ Alex-488 diffusion within an area of less than 10 μm diameters, which was seen under a fluorescent microscope at 488 nm.

### Single channel recordings

VGSCs’ currents were recorded in a cell-attached configuration by MultiClamp-700B and pClamp-10 at the axonal bleb and soma of identical pyramidal neurons. Seal resistance was above 10 GΩ, and pipette resistance was 10–12 MΩ. Pipette solution contains (mM) 120 NaCl, 2 MgCl2, 10 HEPES, 30 TEA and 0.1 mibefradil [13]. The threshold potentials for VGSC activation were measured by adding negative voltage-pulses into the recording pipettes.

### Modeling

Computational simulation was achieved in NEURON (v7.0), and the following facts were taken into account. The dynamics of axonal and somatic VGSCs (Figure 6) was introduced into the axon and soma of a neuronal model, respectively, to test the initiation of action potentials [42,73,74]. Other parameters about VGSCs were based on Hodgkin-Huxley kinetics and current reports [7,9,10].

The equations for the Na current were based on the works of Hodgkin and Huxley. Na current was calculated by Ohmic relation:

$$I_{\mathrm{na}}={\bar{g}}_{\mathrm{na}}m^{3}h\left(V_{m}-E_{\mathrm{na}}\right)$$

*m* and *h* were calculated by first order kinetic equations:

$$m^{'}=\frac{\left(m_{\inf}-m\right)}{\tau_{m}}$$

$$h^{'}=\frac{\left(h_{\inf}-h\right)}{\tau_{h}}$$

*m*_*inf*_ and *h*_*inf*_ represented the steady-state value of *m* and *h. τ*_*m*_ and *τ*_*h*_ were time constant. *m*_*inf*_ and *τ*_*m*_ were calculated by following equations:

$$m_{\inf}=\frac{\alpha }{\alpha +\beta }$$

$$\tau_{m}=\frac{1}{\alpha +\beta }$$

In these equations, *α* and *β* were the functions of local membrane potential (Vm), which were calculated by:

$$\alpha \left(V_{m}\right)=\frac{A\left(V_{m}-V_{\mathrm{th}}\right)}{1-e^{-\left(V_{m}-V_{\mathrm{th}}\right)/\kappa }}$$

$$\beta \left(V_{m}\right)=\frac{-A\left(V_{m}-V_{\mathrm{th}}\right)}{1-e^{-\left(V_{m}-V_{\mathrm{th}}\right)/\kappa }}$$

All constants in these equations were shown in Table one. In terms of *h*, *τ*_*h*_ was described analogously to *τ*_*m*_, but *h*_*inf*_ was given directly by:

$$h_{\inf}=\frac{1}{1+e^{\left(V_{m}-V_{\mathrm{th}}\right)/\kappa }}$$

The constants of this equation also are shown in Table 1.

**Table 1:** Parameters for the equations describing VGSCs

**Channels**	**Variable**	**Function**	**A(/ms)**	**V**_**th**_**(mV)**	**K(mV)**
VGSC on soma	m	α	0.182	−28.2	9
		β	0.124	−28.2	9
	h	α	0.0091	−50	5
		β	0.024	−75	5
		h_inf_		−55	6.2
VGSC on axon	m	α	0.182	−35	9
		β	0.124	−35	9
	h	α	0.0091	−50	5
		β	0.024	−75	5
		h_inf_		−59	6.2

The distributions of VGSCs in these two compartments were based on the references [2,3,7,9,10]. VGSCs’ reversal potential was set at 50 mV. For potassium channels, high-voltage-activated K^+^ channels (Kv) and slowly-activated/non-inactivated M-type K^+^ channels (Km) were added into this neuronal model. To have the initiation of sequential spikes, the densities of Kv and Km were 50 and 100 pS/cm^2^ on these two compartments, respectively. The reversal potential for K^+^ channels was set to −77 mV. In addition, cylinder axon was calculated based on 1.6 μm in diameter and 70 μm in length, as well as the soma was 30 μm in diameter. Their passive electrical properties include *C*_m_ = 1 μF/ cm^2^, *R*_m_ = 15000 Ω/cm^2^ and *R*_i_ = 100 Ω/cm. Resting membrane potentials were −71 mV. The simulations were run under 37°C. The time step was 0.025 ms.

### Data analyses

The data were analyzed if the soma and axon had resting membrane potentials negatively more than −63 mV and action potentials above 90 mV. Criteria for the acceptation of each experiment also included less than 5% changes in resting membrane potential, spike magnitude, input and seal resistance during each experiment. The data for VGSCs’ recording was taken into account if seal resistance reached to 10 GΩ. The values of spike threshold, refractory period and VGSC currents are presented as mean ± SE. The statistical analyses and comparisons of the results among the different subcellular compartments are done by paired t-test.

## Competing interests

Authors claim no competing interests.

## Authors’ contributions

RG and NC contribute to the experiments and data analyses. HQ works on the computational simulation. JHW contributes to project design and manuscript writing. All authors have read and approved the final version of manuscript.

## Supplementary Material

Additional file 1: Figure S1 The same inputs to different sites induce different outputs, while the propagation of spikes is faithful. The insets show simultaneous recordings on soma and axon bleb. The red curves are recordings from soma while the blue curves are from axon. **A)** The same long-time step pulse is injected to axon (left panel) and to soma (right panel) separately. The outputs are different, but keep consistent between the two recording sites. More spikes are induced when long-time step pulse is injected to soma (right panel). **B)** The same fluctuated signal is injected to axon (left panel) and to soma (right panel) separately. The outputs are different, but also keep consistent between the two recording sites. In this case, more spikes are induced when fluctuated signal is injected to axon. Click here for file

Additional file 2: Figure S2 Current-voltage relationships of axon and soma. **A)** Membrane potentials of axon (blue) and soma (red) under grade subthreshold pulses to these two sites respectively. Schematic for step currents is at the bottom of the panel. Dotted lines indicate the points which are chose to calculate current-voltage correlation. **B)** Correlations between input currents and membrane potentials of axon (filled circles and blue line) and soma (open circles and red line, n=9). There’s no obvious difference between two lines. Click here for file

Additional file 3: Figure S3 ATX does not significantly influence spike thresholds and refractory periods at the soma of cortical pyramidal neurons. The spike thresholds and refractory periods are measured dynamically by changing the patterns of input signals at the soma. **A)** shows threshold stimuli vs. depolarization time at the soma. Compared with the control (red symbols), ATX does not change somatic spike thresholds significantly (green symbols; n=12). **B)** illustrates spike refractory periods vs. depolarization time at the soma. Compared with the control, ATX does not change the refractory periods of somatic spikes (green symbols, n=11). Click here for file

## References

[B1] Brock LG Coombs JS Eccles JC Intracellular recording from antidromically activated motoneurones J Physiol Lond 1953 122 429 461 13118553 10.1113/jphysiol.1953.sp005013 PMC1366133

[B2] Clark BA Monsivais P Branco T London M Hausser M The site of action potential initiation in cerebellar Purkinje neurons Nat Neurosci 2005 8 137 139 10.1038/nn1390 15665877

[B3] Colbert CM Pan E Ion channel properties underlying axonal action potential initiation in pyramidal neurons Nat Neurosci 2002 5 533 538 10.1038/nn0602-857 11992119

[B4] Eccles JC The physiology of nerve cells 1957 Baltimore: Johns Hopkins University Press

[B5] Edwards C Ottoson D The site of impulse initiation in a nerve cell of a crustacean sretch receptor J Physiol Lond 1958 143 138 148 13576465 10.1113/jphysiol.1958.sp006049 PMC1356716

[B6] Fuortes MGF Frank K Becker MC Steps in the production of motor neuron spikes J Gen Physiol 1957 40 735 752 10.1085/jgp.40.5.735 13428986 PMC2147645

[B7] Hu W Tian C Li T Yang P Hou H Shu YS Distinct contribution of Nav1.6 and Nav1.2 in action potential initiation and backpropagation Nat Neurosci 2009 12 996 1002 10.1038/nn.2359 19633666

[B8] Kandel ER Spencer WA Brinley FJJ Electrophysiology of hippocampal neuron. I. Sequential invasion and synaptic organization J Neurophysiol 1961 24 225 242 13751136 10.1152/jn.1961.24.3.225

[B9] Kole MHP Ilschner SU Kampa BM Williams SR Ruben PC Stuart GJ Action potential generation requires a high sodium channel density in the axon initial segment Nat Neurosci 2008 11 178 186 10.1038/nn2040 18204443

[B10] Kole MH Stuart GJ Is action potential threshold lowest in the axon? Nat Neurosci 2008 11 1253 1255 10.1038/nn.2203 18836442

[B11] Chen WR Midtgaard J Shepherd GM Forward and backward propagation of dendritic impulses and their synapstic control in mitral cells Science 1997 278 463 467 10.1126/science.278.5337.463 9334305

[B12] Chen WR Shen GY Shepherd GM Hines ML Midtgaard J Multiple modes of action potential initiation and propagation in mitral cell primary dendrite J Neurophysiol 2002 88 2755 2764 10.1152/jn.00057.2002 12424310

[B13] Chen N Yu J Qian H Ge R Wang JH Axons amplify somatic incomplete spikes into uniform amplitudes in mouse cortical pyramidal neurons PLoS One 2010 5 7 e11868 10.1371/journal.pone.0011868 20686619 PMC2912328

[B14] Gasparini S Migliore M Magee JC On the initiation and propagation of dendritic spikes in CA1 pyramidal neurons J Neurosci 2004 24 11046 11056 10.1523/JNEUROSCI.2520-04.2004 15590921 PMC6730267

[B15] Ge R Qian H Wang JH Physiological synaptic signals initiate sequential spikes at soma of cortical pyramidal neurons Mol Brain 2011 4 19 10.1186/1756-6606-4-19 21549002 PMC3113741

[B16] Gulledge AT Stuart GJ Action potential initiation and propagation in layer 5 pyramidal neurons of the rat prefrontal cortex: absence of dopamine modulation J Neurosci 2003 23 11363 11372 14673000 10.1523/JNEUROSCI.23-36-11363.2003 PMC6740518

[B17] Hanson JE Smith Y Jaeger D Sodium channels and dendritic spike initiation at excitatory synapses in globus pallidus neurons J Neurosci 2004 24 329 340 10.1523/JNEUROSCI.3937-03.2004 14724231 PMC6729996

[B18] Larkum ME Waters J Sakmann B Helmchen F Dendritic spikes in apical dendrites of neocortical layer 2/3 pyramidal neurons J Neurosci 2007 27 8999 9008 10.1523/JNEUROSCI.1717-07.2007 17715337 PMC6672209

[B19] Luscher HR Larkum ME Modeling action potential initiation and back-propagation in dendrites of cultured rat motoneurons J Neurophysiol 1998 80 715 729 9705463 10.1152/jn.1998.80.2.715

[B20] Roberts CB Campbell RE Herbison AE Suter KJ Dendritic action potential initiation in hypothalamic gonadotropin-release hormone neurons Endocrinology 2008 149 3355 3360 10.1210/en.2008-0152 18403488 PMC2453095

[B21] Stuart GJ Schiller J Sakmann B Action potential initiation and propagation in rat neocortical pyramidal neurons J Physiol Lond 1997 505 617 632 10.1111/j.1469-7793.1997.617ba.x 9457640 PMC1160040

[B22] Deqenetais E Thierry AM Glowinski J Gioanni Y Electrophysiological properties of pyramidal neurons in the rat prefrontal cortex: an in vivo intracellular recording study Cereb Cortex 2002 12 1 16 10.1093/cercor/12.1.1 11734528

[B23] Haider B Duque A Hasenstaub A McCormick DA Neocortical network activity in vivo is generated through a dynamic balance of excitation and inhibition J Neurosci 2006 26 4535 4545 10.1523/JNEUROSCI.5297-05.2006 16641233 PMC6674060

[B24] Henze DA Buzsaki G Action potential threshold of hippocampal pyramidal cells in vivo is increased by recent spiking activity Neuroscience 2001 105 121 130 10.1016/S0306-4522(01)00167-1 11483306

[B25] Zhang Z Yu YQ Liu CH Chan YS He J Reprint of “frequency tuning and firing pattern properties of auditory thalamic neurons: an in vivo intracellular recording from the guinea pig” Neuroscience 2008 154 273 282 10.1016/S0306-4522(08)00741-0 18555163

[B26] Colbert CM Johnston D Axonal action-potential initiation and Na + channel densities in the soma and axon initial segment of subicular pyramidal neurons J Neurosci 1996 16 6676 6686 8824308 10.1523/JNEUROSCI.16-21-06676.1996 PMC6579266

[B27] Zonta B Desmazieres A Rinaldi A Tait S Sherman DL Nolan MF Brophy PJ A critical role for Neurofascin in regulating action potential initiation through maintenance of the axon initial segment Neuron 2011 69 945 956 10.1016/j.neuron.2011.02.021 21382554 PMC3057015

[B28] Milescu LS Yamanishi T Ptak K Smith JC Kinetic properties and functional dynamics of sodium channels during repetitive spiking in a slow pacemaker neuron J Neurosci 2010 30 12113 12127 10.1523/JNEUROSCI.0445-10.2010 20826674 PMC2945634

[B29] Grubb MS Burrone J Activity-dependent relocation of the axon initial segment fine-tunes neuronal excitability Nature 2010 465 1070 1074 10.1038/nature09160 20543823 PMC3196626

[B30] Kuba H Oichi Y Ohmori H Presynaptic activity regulates Na(+) channel distribution at the axon initial segment Nature 2010 465 1075 1078 10.1038/nature09087 20543825

[B31] Fellous JM Houweling AR Modi RH Rao RPN Tiesinga PHE Sejnowski TJ Frequency dependence of spike timing reliability in cortical pyramidal cells and interneuron J Neurophysiol 2001 85 1782 1787 11287500 10.1152/jn.2001.85.4.1782

[B32] Chen N Chen SL Wu YL Wang JH The refractory periods and threshold potentials of sequential spikes measured by whole-cell recordings Biochem Biophys Res Commun 2006 340 151 157 10.1016/j.bbrc.2005.11.170 16343428

[B33] Chen N Zhu Y Gao X Guan S Wang J-H Sodium channel-mediated intrinsic mechanisms underlying the differences of spike programming among GABAergic neurons Biochem Biophys Res Commun 2006 346 281 287 10.1016/j.bbrc.2006.05.120 16756951

[B34] Mantegazza M Franceschetti S Avanzini G Anemone toxin (ATX II)-induced increase in persistent sodium current: effects on the firing properties of rat neocortical pyramidal neurones J Physiol 1998 507 Pt 1 105 116 9490824 10.1111/j.1469-7793.1998.105bu.x PMC2230778

[B35] Rathmayer W Anemone toxin discriminates between ionic channels for receptor potential and for action potential production in a sensory neuron Neurosci Lett 1979 13 313 318 10.1016/0304-3940(79)91512-X 43492

[B36] Aldrich RW Corey DP Stevens CF A reinterpretation of mammalian sodium channel gating based on single channel recording Nature 1983 306 436 441 10.1038/306436a0 6316158

[B37] Goldman L Stationarity of sodium channel gating kinetics in excised patches from neuroblastoma N1E 115 Eur Biophys 1995 69 2364 2368 10.1016/S0006-3495(95)80105-0 PMC1236473 8599642

[B38] Milescu LS Bean BP Smith JC Isolation of somatic Na + currents by selective inactivation of axonal channels with a voltage prepulse J Neurosci 2010 30 7740 7748 10.1523/JNEUROSCI.6136-09.2010 20519549 PMC2913474

[B39] Melinek R Muller KJ Action potential initiation site depends on neuronal excitation J Neurosci 1996 16 2585 2591 8786434 10.1523/JNEUROSCI.16-08-02585.1996 PMC6578777

[B40] Rush AM Dib-Hajj SD Waxman SG Electrophysiological properties of two axonal sodium channels, Nav1.2 and Nav1.6, expressed in mouse spinal sensory neurones J Physiol 2005 564 803 815 10.1113/jphysiol.2005.083089 15760941 PMC1464456

[B41] Hines ML Carnevale NT The NEURON simulation environment Neural Comput 1997 9 1179 1209 10.1162/neco.1997.9.6.1179 9248061

[B42] Mainen ZF Joerges J Huguenard JR Sejnowski TJ A model of spike initiation in neocortical pyramidal neurons Neuron 1995 15 1427 1439 10.1016/0896-6273(95)90020-9 8845165

[B43] Ge R Chen N Wang JH Real-time neuronal homeostasis by coordinating VGSC intrinsic properties Biochem Biophys Res Commun 2009 387 585 589 10.1016/j.bbrc.2009.07.066 19616515

[B44] Calvin WH Three modes of repetitive firing and the role of threshold time course between spikes Brain Res 1974 69 341 346 10.1016/0006-8993(74)90012-2 4362815

[B45] Hodgkin AL Huxley AF Resting and action potentials in single nerve fibres J Physiol 1945 104 176 195 16991677 10.1113/jphysiol.1945.sp004114 PMC1393558

[B46] Hodgkin AL Beginning: some reminiscences of my early life (1914–1947) Annu Rev Physiol 1983 45 1 16 10.1146/annurev.ph.45.030183.000245 6342510

[B47] Daoudal D Debanne D Long-term plasticity of intrinsic excitability: learning rules and mechanisms Learn Mem 2003 10 456 465 10.1101/lm.64103 14657257

[B48] Desai NS Rutherford L Turrigiano GG Plasticity in the intrinsic excitability of cortical pyramidal neurons Nat Neurosci 1999 2 515 520 10.1038/9165 10448215

[B49] Ganguly K Kiss L Poo M-M Enhancement of presynaptic neuronal excitability by correlated presynaptic and postsynaptic spiking Nat Neurosci 2000 3 1018 1026 10.1038/79838 11017175

[B50] Nelson AB Krispel CM Sekirnjak C du Lac S Long-lasting increases in intrinsic excitability triggered by inhibition Neuron 2003 40 609 620 10.1016/S0896-6273(03)00641-X 14642283

[B51] Nick TA Ribera AB Synaptic activity modulates presynaptic excitability Nat Neurosci 2000 3 142 149 10.1038/72082 10649569

[B52] Sourdet V Russier M Daoudal G Ankri N Debanne D Long-term enhancement of neuronal excitability and temporal fidelity mediated by metabotropic glutamate receptor subtype 5 J Neurosci 2003 23 10238 10248 14614082 10.1523/JNEUROSCI.23-32-10238.2003 PMC6741009

[B53] Spitzer NC Kingston PA Manning TJ JR Conklin MW Outside and in: development of neuronal excitability Curr Opin Neurobiol 2002 12 315 323 10.1016/S0959-4388(02)00330-6 12049939

[B54] Zhang M Hung F Zhu Y Xie Z Wang J Calcium signal-dependent plasticity of neuronal excitability developed postnatally J Neurobiol 2004 61 277 287 10.1002/neu.20045 15382030

[B55] Chen N Chen X Wang J-H Homeostasis established by coordination of subcellular compartment plasticity improves spike encoding J Cell Sci 2008 121 2961 2971 10.1242/jcs.022368 18697837

[B56] Angelides KJ Elmer LW Loftus D Elson E Distribution and lateral mobility of voltage-dependent sodium channels in neurons J Cell Biol 1988 106 1911 1925 10.1083/jcb.106.6.1911 2454930 PMC2115131

[B57] Boiko T Van Wart A Caldwell JH Levinson SR Trimmer JS Matthews G Functional specialization of the axon initial segment by isoform-specific sodium channel targeting J Neurosci 2003 23 2306 2313 12657689 10.1523/JNEUROSCI.23-06-02306.2003 PMC6742039

[B58] Duflocq A Le Bras B Bullier E Couraud F Davenne M Nav1.1 is predominantly expressed in nodes of Ranvier and axon initial segments Mol Cell Neurosci 2008 39 180 192 10.1016/j.mcn.2008.06.008 18621130

[B59] Fleidervish IA Lasser-Ross N Gutnick MJ RW N Na + imaging reveals little difference in action potential-evoked Na + influx between axon and soma Nat Neurosci 2010 13 852 860 10.1038/nn.2574 20543843 PMC3102307

[B60] Garrido JJ Fernandes F Moussif A Fache MP Giraud P Dargent B Dynamic compartmentalization of the voltage-gated sodium channels in axons Biol Cell 2003 95 437 445 10.1016/S0248-4900(03)00091-1 14597261

[B61] Hill AS Nishino A Nakajo K Zhang G Fineman JR Selzer ME Okamura Y Cooper EC Ion channel clustering at the axon initial segment and node of Ranvier evolved sequentially in early chordates PLoS Genet 2008 4 e1000317 10.1371/journal.pgen.1000317 19112491 PMC2597720

[B62] Inda MC Defelipe J Munoz A Voltage-gated ion channels in the axon initial segment of human cortical pyramidal cells and their relationship with chandelie cells Proc Natl Acad Sci U S A 2006 103 2920 2925 10.1073/pnas.0511197103 16473933 PMC1413846

[B63] Lorincz A Nusser Z Cell-type-dependent molecular composition of the axon initial segment J Neurosci 2008 28 14329 14340 10.1523/JNEUROSCI.4833-08.2008 19118165 PMC2628579

[B64] Royeck M Horstmann MT Remy S Reitze M Yaari Y Beck H Role of axonal NaV1.6 sodium channels in action potential initiation of CA1 pyramidal neurons J Neurophysiol 2008 100 2361 2380 10.1152/jn.90332.2008 18650312

[B65] Schmidt-Hieber C Bischofberger J Fast sodium channel gating supports localized and efficient axonal action potential initiation J Neurosci 2010 30 10233 10242 10.1523/JNEUROSCI.6335-09.2010 20668206 PMC6633381

[B66] Van Wart A Trimmer JS Matthews G Polarized distribution of ion channels within microdomains of the axon initial segment J Comp Neurol 2007 500 339 352 10.1002/cne.21173 17111377

[B67] Wollner DA Catterall WA Localization of sodium channels in axon hillocks and initial segments of retinal ganglion cells Proc Natl Acad Sci U S A 1986 83 8424 8428 10.1073/pnas.83.21.8424 2430289 PMC386941

[B68] Wang JH Wei J Chen X Yu J Chen N Shi J The gain and fidelity of transmission patterns at cortical excitatory unitary synapses improve spike encoding J Cell Sci 2008 121 2951 2960 10.1242/jcs.025684 18697836

[B69] Zhou W Goldin AL Use-dependent potentiation of the Nav1.6 sodium channel Biophys J 2004 87 3862 3872 10.1529/biophysj.104.045963 15465873 PMC1304897

[B70] Wang J-H Short-term cerebral ischemia causes the dysfunction of interneurons and more excitation of pyramidal neurons Brain Res Bull 2003 60 53 58 10.1016/S0361-9230(03)00026-1 12725892

[B71] Hori N Tan Y Strominger NL Carpenter DO Intracellular activity of rat spinal cord motoneurons in slices J Neurosci Methods 2001 112 185 191 10.1016/S0165-0270(01)00467-8 11716953

[B72] Chen N Chen X Yu J Wang J-H After-hyperpolarization improves spike programming through lowering threshold potentials and refractory periods mediated by voltage-gated sodium channels Biochem Biophys Res Commun 2006 346 938 945 10.1016/j.bbrc.2006.06.003 16777065

[B73] Kress GJ Dowling MJ Eisenman LN Mennerick S Axonal sodium channel distribution shapes the depolarized action potential threshold of dentate granule neurons Hippocampus 2010 20 558 571 19603521 10.1002/hipo.20667 PMC2975957

[B74] Pospischil M Toledo-Rodriguez M Monier C Piwkowska Z Bal T Fregnac Y Markram H Destexhe A Minimal Hodgkin-Huxley type models for different classes of cortical and thalamic neurons Biol Cybern 2008 99 427 441 10.1007/s00422-008-0263-8 19011929

