# Supplementary figures and images for: Input-dependent subcellular localization of spike initiation between soma and axon at cortical pyramidal neurons
Source: Mol Brain. 2014 Apr 4;7:26. doi: 10.1186/1756-6606-7-26 (PMC4022375; doi:10.1186/1756-6606-7-26)

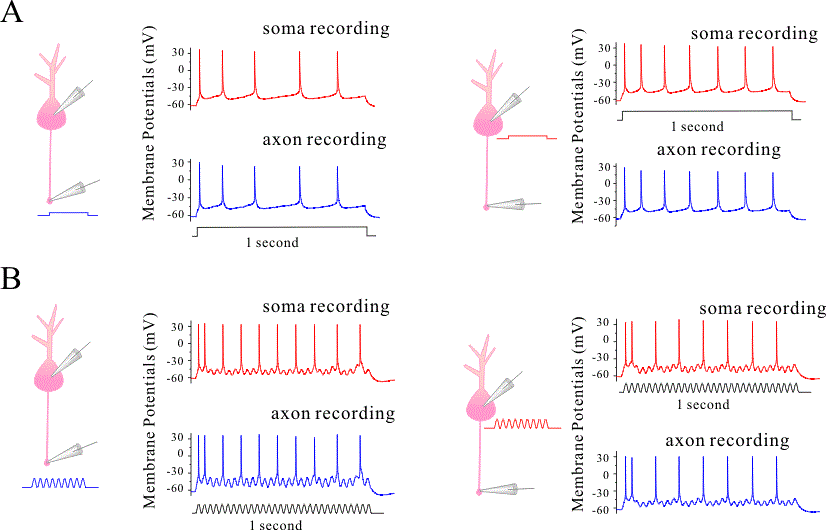

Supplement: Additional file 1: Figure S1 — The same inputs to different sites induce different outputs, while the propagation of spikes is faithful. The insets show simultaneous recordings on soma and axon bleb. The red curves are recordings from soma while the blue curves are from axon. A) The same long-time step pulse is injected to axon (left panel) and to soma (right panel) separately. The outputs are different, but keep consistent between the two recording sites. More spikes are induced when long-time step pulse is injected to soma (right panel). B) The same fluctuated signal is injected to axon (left panel) and to soma (right panel) separately. The outputs are different, but also keep consistent between the two recording sites. In this case, more spikes are induced when fluctuated signal is injected to axon. [file 1756-6606-7-26-S1.tif]

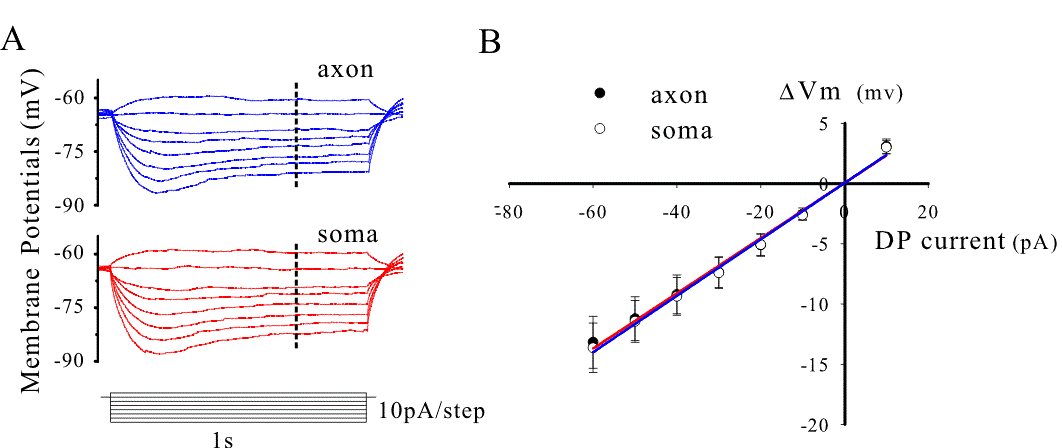

Supplement: Additional file 2: Figure S2 — Current-voltage relationships of axon and soma. A) Membrane potentials of axon (blue) and soma (red) under grade subthreshold pulses to these two sites respectively. Schematic for step currents is at the bottom of the panel. Dotted lines indicate the points which are chose to calculate current-voltage correlation. B) Correlations between input currents and membrane potentials of axon (filled circles and blue line) and soma (open circles and red line, n=9). There’s no obvious difference between two lines. [file 1756-6606-7-26-S2.tif]

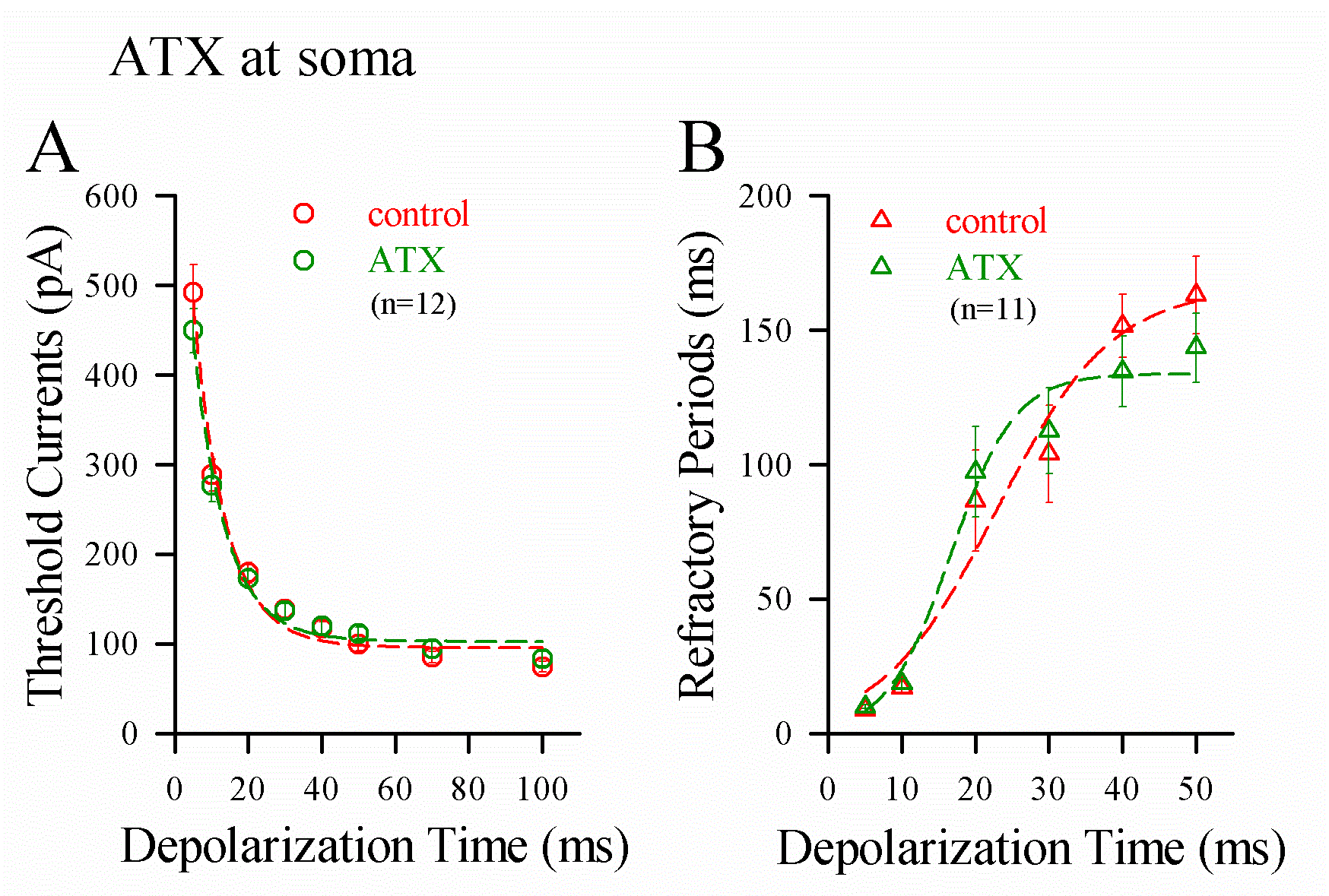

Supplement: Additional file 3: Figure S3 — ATX does not significantly influence spike thresholds and refractory periods at the soma of cortical pyramidal neurons. The spike thresholds and refractory periods are measured dynamically by changing the patterns of input signals at the soma. A) shows threshold stimuli vs. depolarization time at the soma. Compared with the control (red symbols), ATX does not change somatic spike thresholds significantly (green symbols; n=12). B) illustrates spike refractory periods vs. depolarization time at the soma. Compared with the control, ATX does not change the refractory periods of somatic spikes (green symbols, n=11). [file 1756-6606-7-26-S3.tif]
